# Embolization of the first diagonal branch of the left anterior descending coronary artery as a porcine model of chronic trans-mural myocardial infarction

**DOI:** 10.1186/s12967-015-0547-4

**Published:** 2015-06-06

**Authors:** Derek W Hanes, Maelene L Wong, C W Jenny Chang, Sterling Humphrey, J Kevin Grayson, Walter D Boyd, Leigh G Griffiths

**Affiliations:** Department of Veterinary Medicine and Epidemiology, University of California, Davis, One Shields Ave., Davis, CA 95616 USA; University of California Davis, Medical Center, 2221 Stockton Blvd, Sacramento, CA 95817 USA; Clinical Investigation Facility, David Grant USAF Medical Center, 101 Bodin Circle, Travis AFB, CA 94535 USA

**Keywords:** Myocardial infarction, Model, Porcine, D1-LAD, Chronic

## Abstract

**Background:**

Although the incidence of acute death related to coronary artery disease has decreased with the advent of new interventional therapies, myocardial infarction remains one of the leading causes of death in the US. Current animal models developed to replicate this phenomenon have been associated with unacceptably high morbidity and mortality. A new model utilizing the first diagonal branch of the left anterior descending artery (D1-LAD) was developed to provide a clinically relevant lesion, while attempting to minimize the incidence of adverse complications associated with infarct creation.

**Methods:**

Eight Yucatan miniature pigs underwent percutaneous embolization of the D1-LAD via injection of 90 µm polystyrene micro-spheres. Cardiac structure and function were monitored at baseline, immediately post-operatively, and at 8-weeks post-infarct using transthoracic echocardiography. Post-mortem histopathology and biochemical analyses were performed to evaluate for changes in myocardial structure and extracellular matrix (ECM) composition respectively. Echocardiographic data were evaluated using a repeated measures analysis of variance followed by Tukey’s HSD post hoc test. Biochemical analyses of infarcted to non-infarcted myocardium were compared using analysis of variance.

**Results:**

All eight pigs successfully underwent echocardiography prior to catheterization. Overall procedural survival rate was 83% (5/6) with one pig excluded due to failure of infarction and another due to deviation from protocol. Ejection fraction significantly decreased from 69.7 ± 7.8% prior to infarction to 50.6 ± 14.7% immediately post-infarction, and progressed to 48.7 ± 8.9% after 8-weeks (p = 0.011). Left ventricular diameter in systole significantly increased from 22.6 ± 3.8 mm pre-operatively to 30.9 ± 5.0 mm at 8 weeks (p = 0.016). Histopathology showed the presence of disorganized fibrosis on hematoxylin and eosin and Picro Sirius red stains. Collagen I and sulfated glycosaminoglycan content were significantly greater in the infarcted region than in normal myocardium (p = 0.007 and p = 0.018, respectively); however, pyridinoline crosslink content per collagen I content in the infarcted region was significantly less than normal myocardium (p = 0.048).

**Conclusion:**

Systolic dysfunction and changes in ECM composition induced via embolization of the D1-LAD closely mimic those found in individuals with chronic myocardial infarction (MI), and represents a location visible without the need for anesthesia. As a result, this method represents a useful model for studying chronic MI.

**Electronic supplementary material:**

The online version of this article (doi:10.1186/s12967-015-0547-4) contains supplementary material, which is available to authorized users.

## Background

Coronary artery disease and subsequent myocardial infarction (MI) is the leading cause of cardiovascular morbidity and mortality in the United States, accounting for 1 in every 6 deaths [1]. Although advances in MI therapy have improved acute survival rates, survivors are prone to development of chronic degenerative changes as a result of irreversible myocyte loss, extracellular matrix (ECM) fibrosis, and pathological gene programing [2].

Numerous animal models have been developed in an attempt to study the molecular and cellular changes following MI, and to test novel therapies for prevention of chronic negative myocardial remodeling. Historically, dog, sheep, rodent, and rabbit models of MI have been described [3, 4]. More recently, porcine models have gained popularity due to greater similarity in coronary anatomy and cardiac physiology compared to humans. Additionally, the heart-to-body weight ratio of mini-pigs is very similar to that of humans [5]. Further, the larger size of swine, compared to alternative animal models, permits study of clinically relevant therapeutic modalities, such as minimally invasive cardiac interventional procedures. Thus, porcine models have supplanted other animals in the recreation of the native diseases found in humans due to their closer similarity to human cardiac anatomy and pathophysiology.

Previously reported porcine MI models have been associated with substantial challenges, which have limited their utility in assessment of therapeutic candidates. The most commonly used approaches to date have utilized surgical ligation [6–9] or coil embolization [10–12] of the left anterior descending (LAD) coronary artery, distal to the first and/or second diagonal branches as a standard for level of occlusion. Alternatively, ameroid constrictors [5], cryoinjury [13], ethanol infusion [14], autologous platelet embolization [3], balloon occlusion [15–17], and micro-bead LAD embolization [18] procedures have also been described. Regardless of the methodology used to achieve LAD occlusion, serious complications have arisen, including development of malignant ventricular arrhythmias [9, 15, 16], variability of infarct size and location [5, 11], and high mortality rates. Additionally, general anesthesia and expensive imaging modalities, such as magnetic resonance imaging (MRI) [19] or technetium single photon emission tomography (Tc-SPECT) [11] are required to visualize the infarcted territory due to the apical location of the distal LAD territory. Although transthoracic echocardiographic imaging is possible in sedated pigs, the apical location of infarcts produced in distal LAD occlusion models are poorly visualized with this modality. An ideal MI model should include the following criteria: (1) provide consistent lesion size and distribution, (2) result in an infarcted territory in an anatomical location amenable to imaging without the need for anesthesia, and (3) have low mortality rates.

The aims of the current study were to utilize the location of the first diagonal branch of the LAD to create a porcine model of chronic MI that: (1) produces an infarction that models the cellular and molecular changes seen in humans with chronic, trans mural infarcts, (2) produces an infarcted area that can be visualized via standard echocardiography with greater than or equal to 20% reduction in left ventricular ejection fraction (LVEF), and (3) overcomes the adverse complications compared to those reported with previous MI models.

## Methods

This study was performed at an Association for the Assessment and Accreditation of Laboratory Animal Care International-accredited large animal research facility, following approval by the Animal Care and Use Committee at David Grant USAF Medical Center, Travis Air Force Base, California. All animals received care and were used in strict compliance with the Guide for the Care and Use of Laboratory Animals [20].

### Animal inclusion criteria

Eight intact female or castrated male Yucatan mini-pigs were obtained (S & S Farms, Ramona, CA, USA) and acclimated for at least 10 days before use. At the time of surgery, animals weighed between 43 and 66 kg and were approximately 4–5 months of age. All pigs were administered 75 mg clopidogrel bisulfate (Plavix, Bristol-Myers Squibb/Sanofi Pharmaceuticals Partnership, Bridgewater, NJ, USA), 325 mg aspirin, and 600 mg amiodarone hydrochloride (TEVA Pharmaceuticals USA, Sellersville, PA, USA) orally once daily for 2 days prior to, and on the morning of the procedure.

### Anesthesia

Prior to surgery, each animal was fasted for 8–12 h with unlimited access to water. Animals were pre-medicated with 6.6 mg/kg tiletamine/zolazepam (Telazol, Fort Dodge Animal Health, Fort Dodge, IA, USA) intramuscularly (IM). Following isoflurane induction and endotracheal intubation, balanced anesthesia was maintained with gaseous and intravenous agents. The gaseous anesthetic consisted of 1% sevoflurane in 100% oxygen [17]. Animals were mechanically ventilated with tidal volumes of 7–10 mL/kg and a respiratory rate of 10–15 breaths per minute sufficient to maintain end tidal CO_2_ at 40 ± 5 mm Hg. Partial intravenous (IV) anesthesia was also performed using a composite mixture of 0.1 µg/kg/min remifentanil hydrochloride (Ultiva, Mylan Institutional LLC, Rockford, IL, USA), 5 µg/kg/min ketamine hydrochloride (Ketaset, Fort Dodge Animal Health, Fort Dodge, IA, USA), and 0.25 µg/kg/min midazolam hydrochloride (West-Ward Pharmaceutical Corp., Eatontown, NJ, USA) added to 50 µg/kg/min propofol (Diprivan, APP Pharmaceuticals, LLC, Schaumburg, IL, USA) [21, 22]. Two IV catheters were placed in marginal ear veins (or central venous access in the right jugular vein if ear catheterization could not be performed) for administration of fluids. Intravenous fluid administration with 0.9% saline and 5% dextrose in water containing heparin (200–300 U/kg/h) was continued throughout the procedure. Additionally, 1 g cefazolin sodium (West-Ward Pharmaceutical Corp., Eatontown, NJ, USA) was given IV and 2 mg/kg carprofen (Rimadyl, Zoetis, Inc., Kalamazoo, MI, USA) was given IM, prior to beginning the procedure. Animals were monitored during general anesthesia and in the immediate post-operative recovery period using continuous electrocardiography, direct blood pressure, end-tidal CO_2_, inspired and expired inhalant anesthesia, SpO_2_, and temperature.

### Anti-arrhythmic/anticoagulant therapy

Intravenous amiodarone hydrochloride (Mylan Institutional LLC, Rockford, IL, USA) was administered as a 10–12 mg/kg bolus at the start of the procedure and maintained at 8–50 µg/kg/min throughout [4, 23]. Lidocaine hydrochloride (Hospira Inc., Lakeforest, IL, USA) was administered as a 1–2 mg/kg bolus IV at the start of the procedure and continued as an intravenous infusion at 50–200 µg/kg/min during anesthesia. Atropine sulfate (West-Ward Pharmaceutical Corp., Eatontown, NJ, USA) was given at 0.01–0.05 mg/kg as needed for bradycardia.

Heparin sodium (Fresenius Kabi USA, Inc., Schaumburg, IL, USA) was given as a 200–300 U/kg bolus IV at the start of the procedure and continued at a rate of 50–150 U/kg/h IV throughout. Activated clotting time (ACT) was assessed prior to heparin administration, followed by every 30–45 min thereafter, with a goal of maintaining ACT >300 s.

### Myocardial infarction model

Animals were placed in dorsal recumbency and the inguinal area was aseptically prepared. The right femoral artery was identified, and a 21 G, 7 cm echogenic introducer needle (Arrow Intl.; Reading, PA, USA) was placed in the right femoral artery under ultrasound guidance. A 0.018″ nitinol wire (AngioDynamics; Queensbury, NY, USA) was introduced through the needle. The introducer needle was removed and the wire exchanged for a 0.035″ guide wire using a standard micro-puncture sheath (AngioDynamics). A 7 Fr introducer sheath (Arrow Intl.) with side-port was advanced over the guide wire and sutured into position using 2-0 silk suture (Ethicon Inc., Somerville, NJ, USA). Blood pressure was directly monitored via a transducer connected to the side-port throughout the procedure.

**Figure 1:**
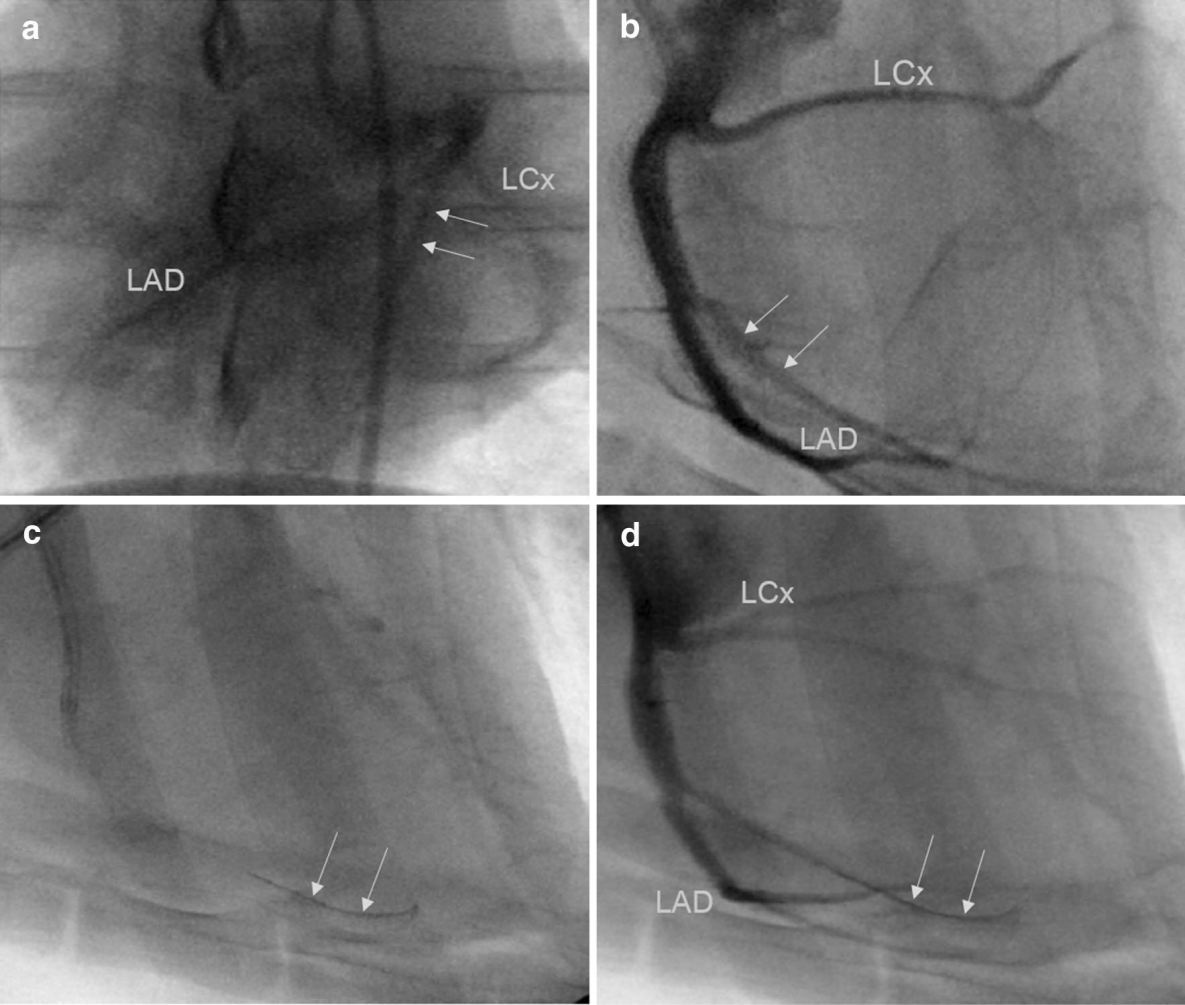
Left main coronary angiography and D1-LAD catheterization. Coronary angiography of the left main coronary artery in ventrodorsal (**a**) and lateral (**b**) projections. The left coronary anatomy, including left circumflex coronary (LCx) and left anterior descending (LAD) arteries is clearly visualized. The D1-LAD artery was identified and is shown outlined here by the *arrows* in the ventrodorsal and lateral views. Following identification of the D1-LAD artery, the artery was then catheterized with a 0.014″ micro-guide wire with correct wire positioning designated by *arrows* on lateral projection (**c**). Repeat angiography was performed following wire advancement (**d**) with the wire still present designated by the *arrows*.

A 6 Fr balloon wedge pressure catheter (Arrow Intl.) was introduced into the femoral artery and the balloon inflated. The catheter was advanced, under fluoroscopic guidance (OEC 9900 Elite; GE Healthcare; Princeton, NJ, USA), to the level of the descending thoracic aorta. A 0.035″ J-tipped exchange wire (Cook Medical; Bloomington, IN, USA) was advanced through the balloon wedge pressure catheter to the level of the aortic arch and advanced into the common carotid artery, and the balloon catheter removed over-the-wire. A 6 Fr H-Stick catheter (Cordis VistaBrite; Miami Lakes, FL, USA) was advanced over-the-wire to the level of the ascending aortic arch and the exchange wire removed. The tip of the catheter was advanced into the ascending aorta and placed in the left coronary ostium. A coronary angiogram was performed by injection of 10 mL of dilute iodixanol (Visipaque; GE Healthcare, Princeton, NJ, USA), diluted 50:50 with sterile saline, into the left main coronary artery (Figure 1). The H-stick catheter was advanced into the proximal LAD artery and angiography repeated to confirm the location of the first diagonal branch of the LAD (D1-LAD) using both ventrodorsal and lateral projections (Figure 1a, b, respectively). Nitroglycerin in 5% dextrose solution (Baxter Healthcare Corporation, Deerfield, IL, USA) was administered in 40 µg boluses through the H-stick catheter to prevent coronary vasospasm as needed. A 0.014″ micro guide-wire (Cook Medical) was advanced through the H-Stick catheter and directed into the D1-LAD branch. Following placement of the micro guide-wire (Figure 1c), angiography was repeated to confirm placement in the D1-LAD (Figure 1d). A 2 mm coronary balloon catheter (Abbott Vascular; Santa Clara, CA, USA) was advanced over-the-wire into the proximal portion of the D1-LAD artery. The balloon catheter was inflated to 6–8 psi and occlusion of the artery was confirmed by S-T segment elevation on continuous electrocardiography. Two mL of 90 µm diameter polystyrene micro-beads (Polybead, Polysciences, Inc., Warrington, PA, USA) was administered through the coronary balloon catheter. Following micro-bead delivery, the balloon catheter was deflated and removed. Angiography was performed to confirm obstruction of the D1-LAD and the remaining catheters removed. The introducer sheath was also removed and hemostasis achieved using manual compression of the femoral artery.

### Echocardiography

**Figure 2:**
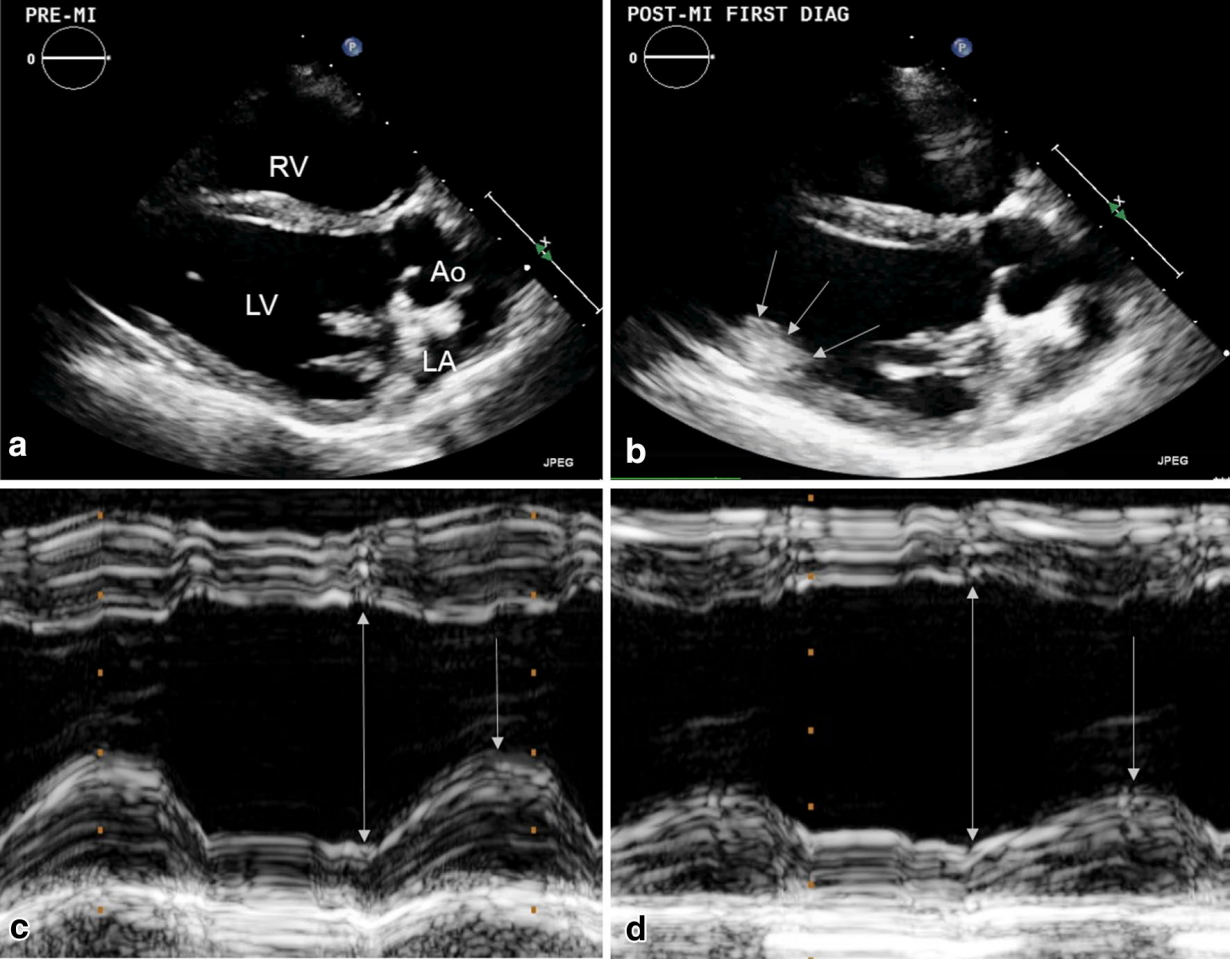
Two-dimensional (2D) and M-mode echocardiography prior to and following D1-LAD occlusion. Echocardiography was performed prior to (**a**) and following D1-LAD occlusion. Occasionally, the presence of the infarct could be seen as a hyperechoic amorphous mass on 2D images, associated with the left ventricular posterior wall and anterolateral papillary muscle, shown here outlined by *arrows* (**b**). Compared to pre-MI M-mode images (**c**), there was an appreciable decrease in systolic excursion of the left ventricular posterior wall following D1-LAD occlusion (**d**). The *double arrows* represent the left ventricle at its maximal diastolic diameter, while the *single arrows* represent the maximal systolic excursion of the LV posterior wall.

All echocardiography was performed at the right parasternal location according to standard imaging techniques, [24] with a S5-1 linear probe (iE33 xMatrix Ultrasound, Philips Healthcare, Andover, MA, USA). Animals were imaged in dorsal recumbency, under anesthesia (during MI) or heavy sedation (at 8-weeks post-MI). A standard left ventricular outflow tract view was used for both 2-D and M-mode assessment (Figure 2). All left ventricular dimensions were measured from M-mode images with the cursor perpendicular to the interventricular septum at the level of the chordae (Figure 2c, d). Both 2D and M-mode images were obtained before and immediately after infarction, as well as at the 8-week endpoint. Three consecutive heart cycles were measured for each patient and all measurements averaged. LVEF was then calculated and expressed as a percentage.

### Post-operative course

Postoperatively, pigs were assessed daily by veterinary staff and were given 2–4 mg/kg carprofen (Rimadyl, Pfizer Animal Health, New York, NY, USA) orally twice daily for 2–3 days. Animals were subsequently monitored for any clinical signs of progression to congestive heart failure over the 8-week study period.

### Gross necropsy and histopathology

Pigs were euthanized at 8-weeks post-MI with an overdose of pentobarbital/phenytoin solution (Beuthanasia-D Special, Schering-Plough Animal Health Corp; Union, NJ, USA). The heart was extirpated, and digital images were taken for later assessment of infarct size and location. The heart was subsequently grossly sectioned to evaluate distribution of the infarcted region and wall thickness compared to non-infarcted myocardium. Sectioned heart tissue samples were stored in 10% buffered formalin solution for 48 h followed by storage in 70% ethanol prior to paraffin embedding and histologic staining. Representative regions of infarcted myocardium were thin-sectioned and stained using hematoxylin and eosin stain to evaluate tissue architecture and composition. Picro Sirius red stain was used to highlight collagen deposition associated with post-MI fibrosis. Verhoeff-Van Gieson (VVG) stained sections were also evaluated in order to assess changes in ECM composition at the infarcted region compared to normal myocardium.

### Biochemical composition analysis

All chemicals were purchased from Sigma-Aldrich (St. Louis, MO, USA) unless otherwise stated. Circumferentially oriented strips (approximately 1 cm × 1 cm × 7 cm) of porcine myocardium were taken from the left ventricular posterior wall and septum of each animal. A slice (approximately 3 mm × 1 cm × 1 cm) from the endocardial to epicardial wall of each strip was then trimmed into a set of four pieces (approximately 2 mm × 3 mm × 1 cm), with each piece containing both endocardial and epicardial surfaces. Following determination of wet weight, samples were frozen at −20°C overnight and lyophilized for 72 h before determination of dry weight. Lyophilized samples were processed accordingly for quantitative biochemistry (*n* = 5 per group and assay).

Hydrochloric acid (5N) was added to one set of lyophilized samples at 200 µL per 10 mg wet weight (WW). Samples were incubated at 100°C for 36 h. Collagen content was quantified using a hydroxyproline assay kit (Chondrex Inc., Redmond, WA, USA) with a calibration curve of 5N hydrochloric acid digested porcine collagen I standards (Chondrex, Inc.). Pyridinoline crosslink content was determined on high performance liquid chromatography (HPLC) using a calibration curve of pyridinoline standards (Quidel, San Diego, CA, USA) as described previously [25].

Papain extraction reagent (0.2 M sodium phosphate buffer, pH 6.4 containing 0.1 M sodium acetate, 0.01 M Na_2_EDTA, 0.005 M cysteine HCl, and 0.4 mg/mL papain) was added to one set of lyophilized samples at 200 µL per 10 mg dry weight (DW). Samples were incubated at 65°C and 1,000 rpm for 24 h three times. Sulfated glycosaminoglycan (GAG) content was quantified from pooled supernatant using the Blyscan sulfated GAG assay (Biocolor Ltd., Carrickfergus, UK) and a calibration curve of GAG standards.

### Statistical analysis

Echocardiographic data was tabulated and compared for each animal at each time point using a repeated measures analysis of variance (ANOVA). Values showing a significant difference were subsequently analyzed using Tukey’s HSD post hoc test. Quantitative biochemical data for infarcted myocardium was compared to septum for each animal using Fit Least Squares. When differences were determined to be significant, Least Squares Means Students *t* test was performed. All other quantitative variables were compared between septum and infarcted myocardium using analysis of variance (ANOVA). Calculations were performed using JMP Pro 11 statistical software (SAS Inst. Inc.) with statistical significance defined as p < 0.05.

## Results

### Viability and myocardial infarction reproducibility

All eight animals were anesthetized and successfully underwent echocardiography prior to percutaneous catheterization. The right femoral artery was successfully catheterized in all animals and the D1-LAD accessed. One pig developed a coronary artery dissection following catheterization and was euthanized under anesthesia, resulting in an 87.5% (7/8 pigs) acute procedural survival rate. Micro-bead delivery was not achieved in one pig due to clot formation in the balloon catheter. Consequently, this pig was excluded from echocardiographic data analysis. Ultimately, a total of 6 of 8 pigs (75%) were successfully embolized and included in the post-embolization evaluation (n = 6). One pig died due to a deviation from protocol during the post-anesthesia recovery period, resulting in a total of five pigs (n = 5) for the chronic MI (8-week) comparison. Overall, procedural success rate at 8-weeks was therefore 71% (5/7 pigs) with one pig censored due to deviation from post-anesthetic recovery protocol. Overall procedural survival rate was 83% (5/6 pigs), with one pig censored due to deviation in post-anesthetic recovery protocol and another censored due to failure of micro-bead delivery. No clinical signs of congestive heart failure were expressed by any of the animals during the subsequent 8-weeks. Procedural time was 117.5 ± 6.5 min.

### Echocardiography

**Figure 3:**
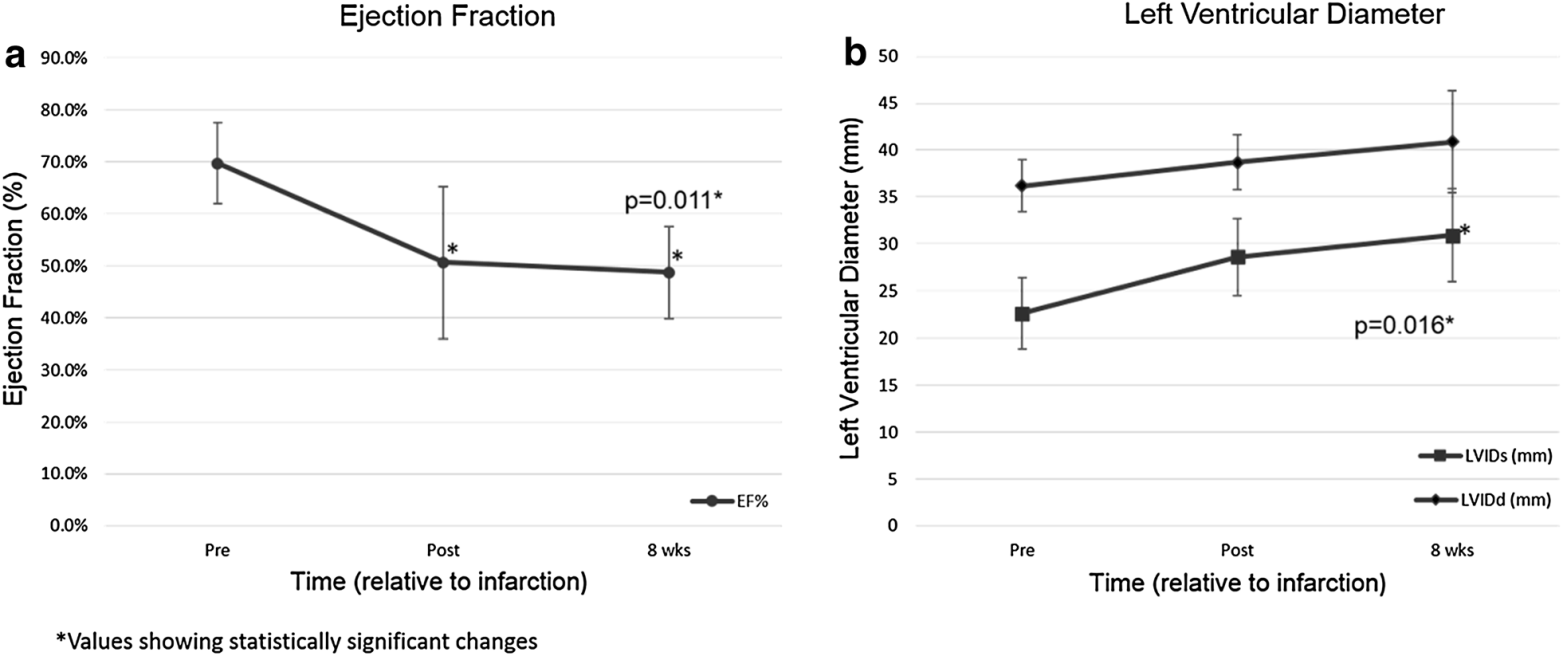
Changes in left ventricular ejection fraction (LVEF) and systolic internal diameter (LVIDs). LVEF decreased immediately following embolization of the D1-LAD artery (**a**). This decrease in LVEF continued through the 8-week endpoint (**a**). At 8-weeks post-embolization LVIDs was significantly increased compared to baseline (**b**). There was an apparent trend to LVIDd enlargement as well throughout the allotted timeframe.

In all successfully infarcted pigs, left ventricular posterior wall and papillary muscle motion was subjectively decreased on 2-D echocardiographic assessment immediately following embolization (Figure 2c, d). In some cases, the micro-bead embolus was visualized as a hyperechoic amorphous region in the left ventricular posterior wall and anterolateral papillary muscle (see Additional file 1). LVEF significantly decreased from 69.7 ± 7.8% prior to infarction to 50.6 ± 14.7% immediately post-MI, and progressed to 48.7 ± 8.9% (30% decrease from baseline) at the 8-week end-point (p = 0.011) (Figure 3a). Left ventricular internal diameter in systole (LVIDs) was significantly increased from 22.6 ± 3.8 mm pre-operatively to 30.9 ± 5.0 mm at 8-weeks post-MI (p = 0.016) (Figure 3b). There was also an increasing trend in left ventricular internal diameter during diastole (LVIDd) from 36.2 ± 2.8 mm prior to infarction to 38.7 ± 2.9 mm and 40.9 ± 5.4 mm following infarction and at 8-weeks respectively (Figure 3b), though this parameter did not reach statistical significance (p = 0.15). No significant changes were appreciated in other ventricular parameters over the allotted timeframe.

### Gross evaluation and histopathology

**Figure 4:**
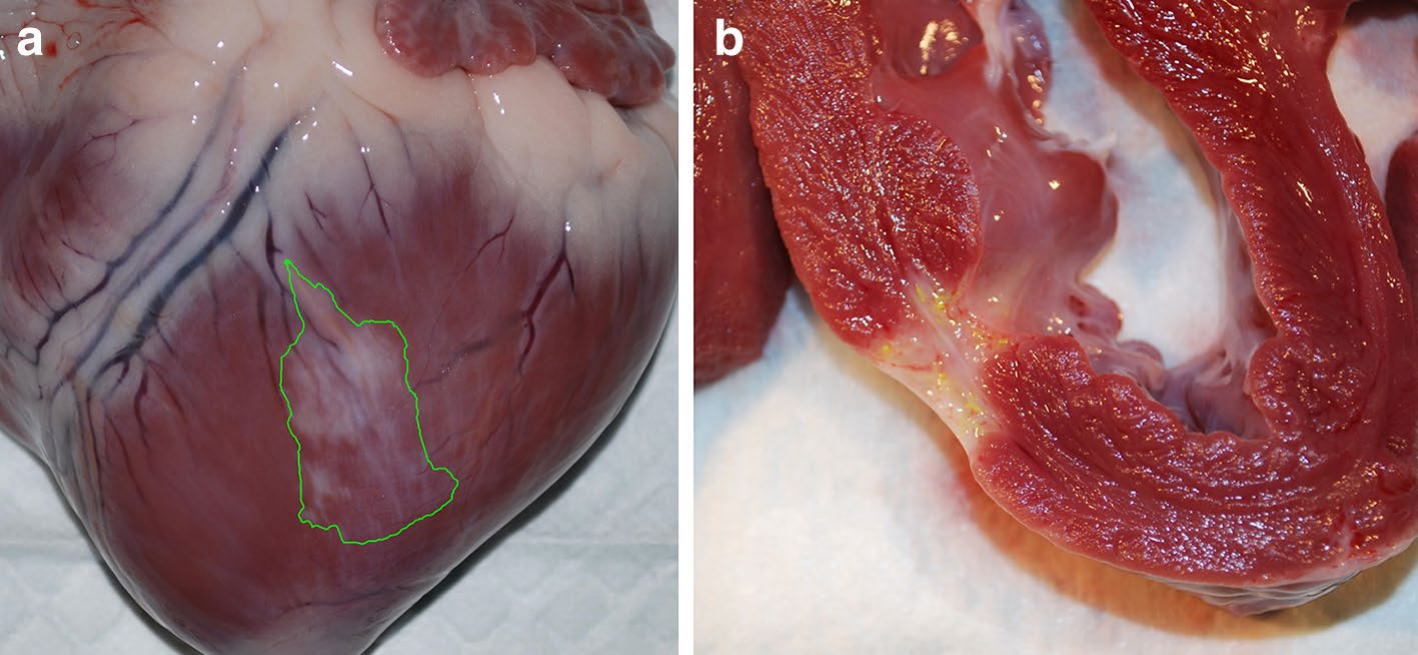
Gross cardiac evaluation of infarct size and distribution. Gross cardiac evaluation at 8 weeks post-MI showed a multifocal, pale, light tan, firm area in the left ventricular posterior wall, midway between the apex and left circumflex coronary artery (*arrows*) (**a**). Transverse sections of the heart showed that the infarcted area extended into the full thickness of the myocardium with subsequent ventricular wall thinning when compared to the adjacent normal myocardium (**b**).

Gross evaluation of the hearts in situ revealed a multifocal, pale white, firm area of infarction associated with the left ventricular posterior wall, midway between the apex and left circumflex coronary artery (Figure 4a). Transverse sections of the heart demonstrated mottled full thickness fibrosis of the left ventricular wall, and wall thinning associated with the infarcted region was observed compared to surrounding normal myocardial tissue (Figure 4b).

**Figure 5:**
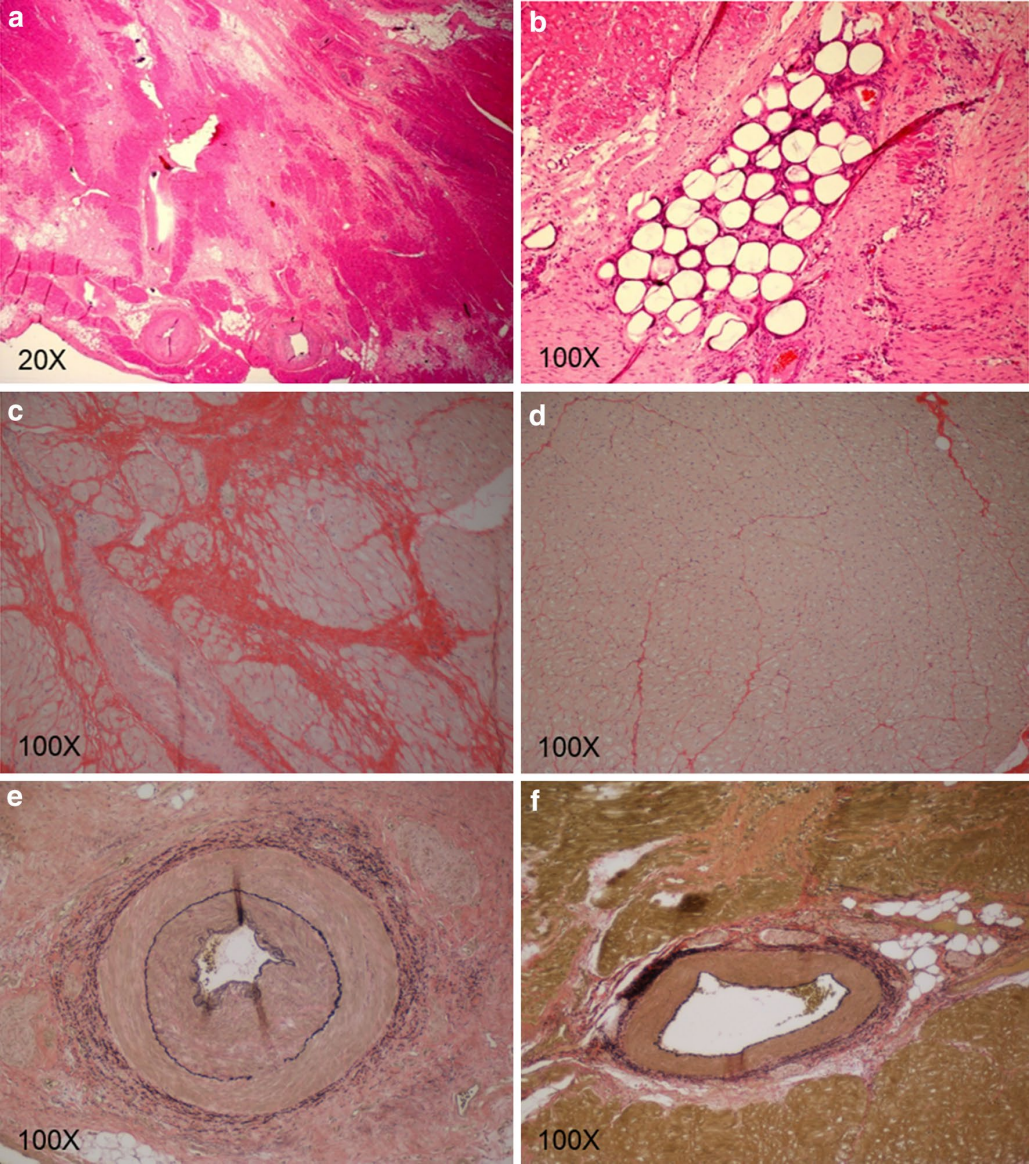
Histopathology of infarcted versus normal myocardium. Hematoxylin and eosin staining of representative infarcted areas demonstrated a heterogenous area of dense, fibrous, connective tissue intermixed with islets of intact cardiomyocytes (**a**). Microsphere silhouettes were visualized occluding small arteries and arterioles shown here as stain filling defects (**b**). Further evaluation with Picro Sirius stain revealed a large amount of disorganized collagen deposition and perifiber fibrosis in the infarcted region denoted by the deeply red staining sections (**c**), when compared to normal myocardium (**d**). Evaluation of VVG stained slides demonstrated hyperplasia of the arterial walls, particularly in the media and adventitial layers (**e**) when compared to normal arteries (**f**).

Hematoxylin and eosin-stained sections demonstrated a heterogenous area of fibrosis and myocytolysis, intermixed with islets of intact cardiomyocytes associated with small arteries and arterioles (Figure 5a). Occasionally, micro-sphere silhouettes could be visualized obstructing small arteries (Figure 5b). Evaluation of Picro Sirius red-stained slides showed a large amount of irregularly oriented, perifiber collagen deposited in the infarcted region (Figure 5c) which was not observed in the adjacent normal myocardium (Figure 5d). Evaluation of VVG-stained sections revealed changes in the arterial composition associated with the infarcted region. Arteries were found to have hyperplastic layering associated with the laminas media and adventitia (Figure 5e) when compared to normal arteries in unaffected myocardium (Figure 5f).

### Biochemical composition analysis

**Figure 6:**
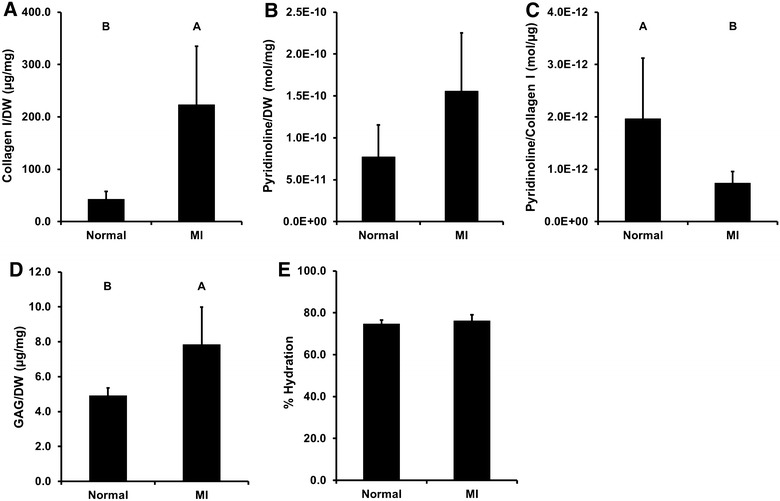
Biochemical composition of normal and infarcted myocardium. Collagen I content was significantly greater in infarcted myocardium than normal septum (**A**). Although pyridinoline crosslink content trended lower in the septum than the MI, the difference was not statistically significant (**B**). Pyridinoline crosslink content per collagen I content was significantly lower in infarcted myocardium than normal septum (**C**). Sulfated GAG content was significantly greater in infarcted myocardium than normal septum (**D**). Water content in MI was not significantly different than that in normal septum (**E**). Results plotted as mean ± standard deviation. Groups not connected by *same letter* are significantly different, p < 0.05 (n = 5 per group).

The water content of infarcted myocardium (76.30 ± 2.84%) was not significantly different from that of normal myocardium (74.74 ± 1.82%) (p = 0.332) (Figure 6E). The collagen I content of infarcted myocardium (223.51 ± 111.13 µg/mg DW) was significantly greater than that of normal myocardium (42.72 ± 14.64 µg/mg DW) (p = 0.007) (Figure 6A). The pyridinoline content of infarcted myocardium (1.56 × 10^−10^ ± 6.93 × 10^−11^ mol/mg DW) was not significantly different than that of normal myocardium (7.75 × 10^−11^ ± 3.79 × 10^−11^ mol/mg DW) (p = 0.058) (Figure 6B). Pyridinoline content per collagen I content was significantly lower in infarcted myocardium (7.38 × 10^−13^ ± 2.18 × 10^−13^ mol/µg) than normal myocardium (1.97 × 10^−12^ ± 1.16 × 10^−12^ mol/µg) (p = 0.048) (Figure 6C). The GAG content of infarcted myocardium (7.84 ± 2.16 µg/mg DW) was significantly greater than that of normal myocardium (4.92 ± 0.43 µg/mg DW) (p = 0.018) (Figure 6D).

## Discussion

Coronary artery disease, leading to myocardial infarction, is estimated to result in 1 of every 6 deaths in the US [1]. Consequently, extensive effort has been committed to researching methods for monitoring and treating MI, including the development and optimization of an animal model to study this disease. However, the associated high degree of morbidity and mortality resulting from malignant ventricular arrhythmias, progression to congestive heart failure, and cardiogenic shock limit the utility of current MI models [3, 14]. Predominant use of the distal LAD as the location of embolism likely contributes to the aforementioned complications, as the LAD supplies a large region of myocardium, including the apex, distal interventricular septum, and a portion of the right ventricle [11, 14]. Since pigs are at elevated risk of death when the ischemic area is greater than 25% of the left ventricle [9], and the LAD perfuses approximately 35% of the left ventricular mass [18], the use of the distal LAD may be responsible for the high mortality associated with MI models created via this location. Additionally, the distal interventricular septum and apex locations are inherently difficult to visualize without the use of expensive equipment such as MRI [11, 14], which carries an additional risk of anesthetic complication and added cost. Given the location of the D1-LAD artery in the left ventricular posterior wall, a new model utilizing the D1-LAD artery as the site of embolism was hypothesized to restrict the infarct location to the left ventricular posterior wall, reducing the variability in infarct size and location, and subsequently, the number of adverse complications. Further, the mid-left ventricular wall can be easily visualized using standard echocardiographic techniques, thus subverting the need for more expensive and less widely available imaging modalities.

Chronic negative remodeling following acute MI is characterized by a progressive reduction in ventricular contractility and compensatory LV remodeling. Ventricular myocytes in the ischemic zone undergo necrosis and myocytolysis, while other cells may activate caspase enzymes and undergo apoptosis as a result of ischemic injury [26]. This myocardial damage ultimately results in decreased systolic function with compensatory eccentric hypertrophy of the surviving myocardium [27], ultimately manifesting as a decrease in LVEF on transthoracic echocardiography [28]. Studies conducted to evaluate the degree with which systolic dysfunction affects prognosis in patients with chronic myocardial infarction found that the severity of contractile dysfunction, represented as a decreased LVEF, was found to be associated with a poorer prognosis for progression to congestive heart failure in a non-linear fashion [28]. The impaired systolic function associated with embolization of the D1-LAD correlates well with surviving patients following infarction. In this model, systolic excursions of the left ventricular posterior wall were visibly decreased resulting in an immediate, statistically significant reduction in LVEF. Utilizing this model for MI, the LVEF was found to not only be reduced immediately following the acute ischemic insult, but continued to decline over the 8-week follow-up period. Alternatively, this can be contrasted to currently used models, which result in an immediate decline in LVEF followed by a rebound [18], or no significant drop in LVEF [10]. Continued decrease in LVEF and systolic function through the 8-week time point suggests that this model resulted in a significant MI lesion, which continued to expand over the time course of the study. The 30% reduction in LVEF achieved represents a clinically relevant lesion size for studying and evaluating MI. Critically, lesion size was also consistently small enough to avoid the complications associated with heart failure or arrhythmogenesis. Through utilization of the D1-LAD artery as the site of embolism, this model was able to produce a clinically relevant reduction in systolic function that mimics that found in the naturally occurring disease, while keeping the lesion confined to a size that did not result in high morbidity or mortality.

In concert with decreasing systolic function, ventricular remodeling results in chamber dilation and eccentric hypertrophy [28]. Decreased systolic function following acute MI activates neurohumoral mechanisms, such as locally produced angiotensin converting enzyme and increased production of angiotensin II [26]. These enzymes initially intended to restore cardiac performance, ultimately result in negative remodeling of the myocardial ECM. This phenomenon, combined with eccentric hypertrophy of the surviving myocardium, results in chamber dilation and progressive impairment in systolic function [28]. These changes can be quantified via transthoracic echocardiography of left ventricular diameter, with progressive increases in LVIDs representing progressive contractile dysfunction and progressive increases in LVIDd representing chamber dilation. In studies evaluating negative prognostic indicators in patients with chronic myocardial infarction, progressive increases in left ventricular end-systolic volume have been correlated with poorer prognosis for progression to heart failure [29]. Indeed, in the D1-LAD model, LVIDs immediately increased following infarction, most likely as a result of stunned myocardium and decreased systolic function (Figure 3) following MI. The increase in LVIDs progressed over the 8-week study period showing sustained and progressive systolic dysfunction utilizing this model. Additionally, there was a trend toward increased LVIDd, representing mild chamber dilation over the course of the study even though this value failed to reach statistical significance. These changes in chamber size are presumably a consequence of the same adverse cardiac remodeling that has been described in humans [28]. The acute and chronic changes in myocardial function demonstrated in this study indicate that D1-LAD embolism results in a clinically relevant MI lesion with subsequent chronic remodeling that mimic human cardiac pathophysiology.

Following initial necrosis and myocytolysis, functional myocardium is replaced with heterogeneous collagen deposition and myofibroblast differentiation [30]. During acute ischemic injury, necrosis and apoptosis result in sub-acute and acute cardiomyocyte death, respectively. Following injury, the cells release signaling factors and cytokines that ultimately result in stimulation of neutrophils and mononuclear cells [26]. Glycosaminoglycans within the ECM facilitate the recruitment of peripheral mononuclear cells by sequestering secreted chemokines [31]. This sterile inflammation results in removal of the damaged myocardial tissue. Subsequent healing of the myocardium is initiated with the stimulation of fibroblast proliferation and collagen deposition. This scar tissue is ultimately less functional contributing to decreases in contractility and increasing wall stress [28]. Humans with coronary artery disease have been shown to have increased perifiber fibrosis and perivascular fibrosis associated with the ischemia found with chronic coronary disease [30]. The D1-LAD MI model resulted in a mottled distribution of infarcted tissue in the LV posterior wall similar to that found in human patients when evaluated via histopathology. On both H&E and Picro Sirius red-stained slides, myocytolysis had been replaced with fibrosis interspersed with islets of intact cardiomyocytes. Indeed, collagen content of the infarct zone was fivefold greater than that of the septum. In patients with end-stage heart failure following MI, collagen content of the scar region was approximately tenfold greater than that of a remote region [32]. Although pyridinoline crosslink content in the infarct zone was not significantly different than that in the septum, the proportion of pyridinoline crosslinks per collagen was two to threefold less in infarcted myocardium than in the septum, suggesting that the collagen associated with the infarct scar was newly synthesized [33] as a result of the chronic negative remodeling following MI. Additionally, the slight elevation in sulfated GAG content of infarcted myocardium above that of septum has been observed following MI by embolization of the D1-LAD. These changes to the ECM in infarcted myocardium correlate well with the changes that occur in humans with chronic myocardial infarctions where myofibers have been replaced with newly deposited immature collagen [30, 34].

Utilizing the D1-LAD artery, monitoring of the infarct location and left ventricular parameters was possible via transthoracic echocardiography, a preferred alternative to more time consuming and expensive methods. Distribution of the LAD territory in the apical region is difficult to visualize via echocardiography, requiring expensive and less widely available imaging modalities. MRI [14] or Tc-SPECT [11] have traditionally been used for monitoring infarct distribution and cardiac function following occlusion of the LAD. Unfortunately, pigs undergoing these modalities must undergo anesthesia for the duration of the scan which is associated with an additional inherent risk. These methodologies are also less widely available, more cost prohibitive, and require longer scan times than transthoracic echocardiography. In this study, all subjects successfully underwent transthoracic echocardiography with subsequent chronic monitoring for systolic function and chamber dilation. The lesion location in the left ventricular posterior wall formed by embolization of the D1-LAD resulted in an embolus location that could be directly visualized in the left ventricular posterior wall (see Additional file 1). Changes on echocardiographic evaluation correlated well with post-mortem findings when evaluating the heart grossly. This is in contrast to other studies that have shown interventricular septal involvement and right ventricular involvement, with subsequent wall thinning through utilization of the distal LAD [8, 14]. Utilization of the D1-LAD artery appears to have restricted the area of infarction to the left ventricular posterior wall, resulting in a less variable and dispersed infarct region that still produced a clinically relevant lesion. Furthermore, the developed D1-LAD embolization methodology was able to create an infarction that could be directly visualized, as well as chronically monitored for progressive systolic dysfunction and chamber dilation, via standard echocardiography.

Development of malignant ventricular arrhythmias causing acute mortality in study subjects has been the most commonly reported complication associated with previously reported porcine MI models [3, 9, 15, 35]. Previously reported LAD occlusion methods resulted in up to 50% [36] procedural attrition rates due to the development of ventricular arrhythmias or cardiogenic shock. The 17% procedural attrition rate with the developed D1-LAD embolization method was due to an arterial dissection upon catheterization of the D1-LAD artery, though it is unclear as to whether this lesion is specific to D1-LAD access, or if such a lesion could occur in any procedure in which catheterization of the LAD is required. Although occasional premature ventricular contractions were detected during these procedures, none of the subjects were lost due to the development of malignant ventricular arrhythmias. Therefore, the developed D1-LAD embolization method results in a low mortality rate, with minimal production of intraoperative ventricular arrhythmias.

Although this methodology appears to have significant utility for research in chronic myocardial infarctions, it is recognized that several study limitations exist. Firstly, catheterization of the D1-LAD artery is somewhat more technically challenging than engaging the distal LAD, requiring greater procedural time and experience to execute. Subsequently, longer anesthetic times and greater risk for complications such anesthetic related complications or arterial dissection may be of higher potential with this procedure than previously reported methods. Additionally, the procedure was most successfully performed in pigs >50 kg where the D1-LAD artery was large enough to be consistently engaged without delay (data not shown). Lastly, embolism in one subject was unsuccessful due to clot formation in the instrumentation, highlighting the importance of adequate anticoagulant therapy for successful bead deployment. In spite of these limitations, embolization of the D1-LAD artery represents a good model for clinical research.

## Conclusions

The porcine chronic MI model using embolization of the D1-LAD described here appears to be a clinically relevant recreation of the functional, architectural, and biochemical changes that occur following ischemic insult in humans. Additionally, the location of the embolism created with this method permits visualization of the MI lesion and monitoring of left ventricular structure and function via widely available imaging modalities in non-anesthetized animals. In the current study, the area of infarction was isolated to the left ventricular wall, and easily visible via standard transthoracic echocardiography. Gross and histopathologic lesion distribution were found to be heterogenous and representative of the pathology clinically documented in human patients with chronic MI. Our findings in this study support a new methodology of creating MI lesion in porcine models which can be easily monitored via echocardiography and closely mimics the pathophysiology associated with human MI patients.

## Additional file

Additional File 1. Left ventricular short axis echocardiographic video loop following embolization (.avi). The location of the infarct can be directly visualized as an amorphous, hyperechoic area associated with the anterolateral papillary muscle and left ventricular wall.

## Abbreviations

D1-LAD: first diagonal branch of the left anterior descending coronary artery
MI: myocardial infarction
ECM: extracellular matrix
LAD: left anterior descending coronary artery
MRI: magnetic resonance imaging
Tc-SPECT: technetium single photon emission tomography
LVEF: left ventricular ejection fraction
IV: intravenous
CO_2_: carbon dioxide
SpO_2_: pulse oximetry
ACT: activated clotting time
H&E: hematoxylin and eosin
VVG: Verhoeff Van Gieson
GAG: glycosaminoglycan
ANOVA: analysis of variance
LVIDs: left ventricular internal diameter in systole
LVIDd: left ventricular internal diameter in diastole
DW: dry weight
WW: wet weight
LCx: left circumflex coronary artery
